# Postoperative cosmetic outcome of intraoperative radiotherapy in comparison to whole breast radiotherapy in early stage breast cancer; a retrospective cohort study

**DOI:** 10.1186/s12885-022-10468-9

**Published:** 2023-01-03

**Authors:** Nahid Nafissi, Seyed Mostafa Meshkati Yazd, Reza Shahriarirad, Saba Zangeneh, Sahar Ghorbani, Borna Farazmand, Mohammadreza Karoobi, Hamid Reza Mirzaei

**Affiliations:** 1 Department of Breast, Rasoul Akram Hospital Clinical Research Development Center (RCRDC), Iran University of Medical Sciences, Tehran, Iran; 2 Department of Surgery, Imam Khomeini Hospital Complex, Tehran University of Medical Sciences, Tehran, 01136746911 Iran; 3 Thoracic and Vascular Surgery Research Center, Shiraz University of Medical Science, Shiraz, Iran; 4 Student Research Committee, Shiraz University of Medical Sciences, Shiraz, Iran; 5 Fasa University of Medical Sciences, Fasa, Iran; 6 Radiation Oncology Research Center, Iran Cancer Institute, Tehran University of Medical Sciences, Tehran, Iran; 7 Cancer Research Center, Shohadae Tajrish Hospital, Department of Radiation Oncology, Shahid Beheshti University of Medical Sciences, Tehran, Iran

**Keywords:** Breast cancer, IOERT, WBR, Cosmetics

## Abstract

**Background:**

In this study, we aim to evaluate the cosmetic outcome differences between Intraoperative electron beam radiation therapy (IOERT) and whole breast radiotherapy (WBR) with further investigation of boosted IOERT.

**Methods:**

This retrospective cohort study was conducted in two referral centers in Tehran, Iran. 116 women aged 30 to 79 with early-stage breast cancer (T0-2N0-1M0) eligible for breast conservation were divided into two groups of 58 based on the intervention they received, and further subgroups were defined based on receiving boosted IOERT. Patients in both groups underwent breast conservation surgery and those in the IOERT group received either a 21 Gy radical dose (radical IOERT) or 12 Gy boosted electron beam radiotherapy and a routine fractionated dose of 50 Gy in 25 sessions of WBR (boosted IOERT). Those in the WBR group were administered 50Gy in 32 sessions. Physician-assessed cosmetic outcome was defined as the primary result and incidence of fat necrosis and fibrosis and post-operative chronic pain were secondary outcomes.

**Results:**

Post-operative cosmetic outcome scores and chronic pain, showed no significant difference between the two groups. The median cosmetic score in both groups was 9. Fat necrosis and fibrosis had significantly higher rates in the IOERT group (P. Value: 0.001). However, the majority (21/34 or 61.8%) of this complication was observed in the boosted IOERT subgroup and no statistical significance was recorded between the radical IOERT subgroup and the WBR group.

**Conclusions:**

In early-stage breast cancer treatment, radical IOERT has noninferiority compared to WBR in terms of cosmesis. Regarding fat necrosis and fibrosis, boosted IOERT was associated with higher rates in comparison to other groups. Therefore, radical IOERT seems to be a better treatment option for selected patients.

## Background

Breast-conserving surgery is the main course of treatment for early-stage breast cancer which consist of local resection of a tumor while retaining the basic shape of the breast [1]. It has been stated that this method combined with postoperative radiation therapy is associated with approximately the same disease-free survival and overall survival rates compared with total mastectomy [2]. However, this multimodality approach (known as breast-conserving therapy (BCT)) also improves patients’ quality of life [3]. Adjuvant radiation therapy in the form of whole breast radiotherapy (WBR) is delivered in 31–32 sessions and a booster dose may be added to the treatment course. The goal of this radiotherapy is to eliminate the residual disease and reduce the recurrence of cancer after surgery [4]. Although this method seems effective in terms of disease control, there are some disadvantages mentioned such as complex tumor margin and considerable differences among individuals making uniform distribution of the radiation a challenge, and [5] approximation of the location and depth of tumor bed may cause errors in determining the appropriate energy of the radiation. Furthermore, breathing pattern and the level of lung expansion seems to be of great importance in the dosimetry of the radiation which may compromise adjacent anatomical structures [6]. Another major problem with WBR is that the long course of treatment along with the lack of access to radiotherapy centers, specifically for the rural population, might discourage them from finishing their radiotherapy treatment [7].

Accelerated partial breast irradiation (APBI) involves delivering a larger dosage of radiation over a shorter length of time to a smaller volume of breast tissue [8]. Intraoperative electron beam radiation therapy (IOERT) is a form of APBI which delivers accelerated single-dose irradiation after lumpectomy to the residual breast tissue during the same anesthetic period. This method is associated with certain advantages including minimal treatment-related toxicities since the irradiation is focused on a smaller volume of the breast [9], theoretically may increase utilization of breast conservation surgery [10] and since the radiotherapy duration is shorter, it will be easier to integrate with chemotherapy schedule and reduce overall treatment duration. However, certain limitations such as not being an optimal treatment option for young patients and patients with large tumors or tumors located near the skin, have been mentioned [11]. Other potential disadvantages of this method are late toxicity, a slightly increased risk of local recurrence, and debatable cosmetic outcomes in comparison to external beam radiation therapy [12].

When presenting therapy choices to breast cancer patients, practitioners must be aware of the aesthetic outcomes and complications associated with each method of treatment, especially when comparing therapies with equivalent efficacy and survival rates. Therefore, in this cohort study, we aim to evaluate and compare cosmetic outcomes and common postoperative complications between intraoperative electron radiotherapy and whole breast radiotherapy.

## Methods

### Study setting

This is a retrospective cohort study that was approved by the Research Ethics Committee of the School of Medicine-Tehran University of Medical Sciences (Ethics code: IR.IUMS.REC.1400.77). The purpose of this study was to evaluate the cosmetic outcome in breast cancer patients receiving IOERT in comparison to those who received whole breast radiotherapy. The study population was collected retrospectively from September 2013 to April 2018 from two tertiary referral centers for breast cancer; Khatam-al-Anbya Hospital and Rasoul Akram Hospital affiliated with the Iran University of Medical Sciences, Tehran, Iran. All surgical procedures in both hospitals were performed by one surgeon.

### Participants

The eligibility criteria for the study population were women with histologically or cytologically proven early-stage breast cancer. The early stage was defined as tumors classified as American Joint Committee on Cancer (AJCC) stage T0-2N0-1M0 [13]. Patients with missing clinical data were omitted from the study. One hundred and sixteen patients with early-stage breast cancer were included. Patients were divided into two groups intraoperative electron beam radiation therapy (IOERT) and whole breast radiotherapy (WBR), each with fifty-eight participants.

The inclusion criteria for the IOERT group in this study were according to GEC-ESTRO and SSO-ASTERO criteria for IOERT [14, 15]. Patients with an extensive intraductal component, diffuse malignant microcalcifications, patients with a history of collagen vascular diseases, and patients in early or midterm pregnancy or breastfeeding were excluded from the study.

### Intervention

Surgical incision and operation type were similar in both groups to reduce heterogeneity between the two groups for comparing cosmetic outcomes. Patients in both groups underwent a lumpectomy and the margin of tumoral tissue and sentinel node were excised and sent as a frozen section for pathological examination. Following that, fibroglandular tissue was released to rotate in tumor defect as an oncoplastic flap. In the IOERT group, these flaps were sutured on a protective disk according to the size of resection and then the IOERT applicator was used to cover marginal tissue completely before radiation, and an electron beam radiated the tissue near 1–2 minutes.

**Fig. 1:**
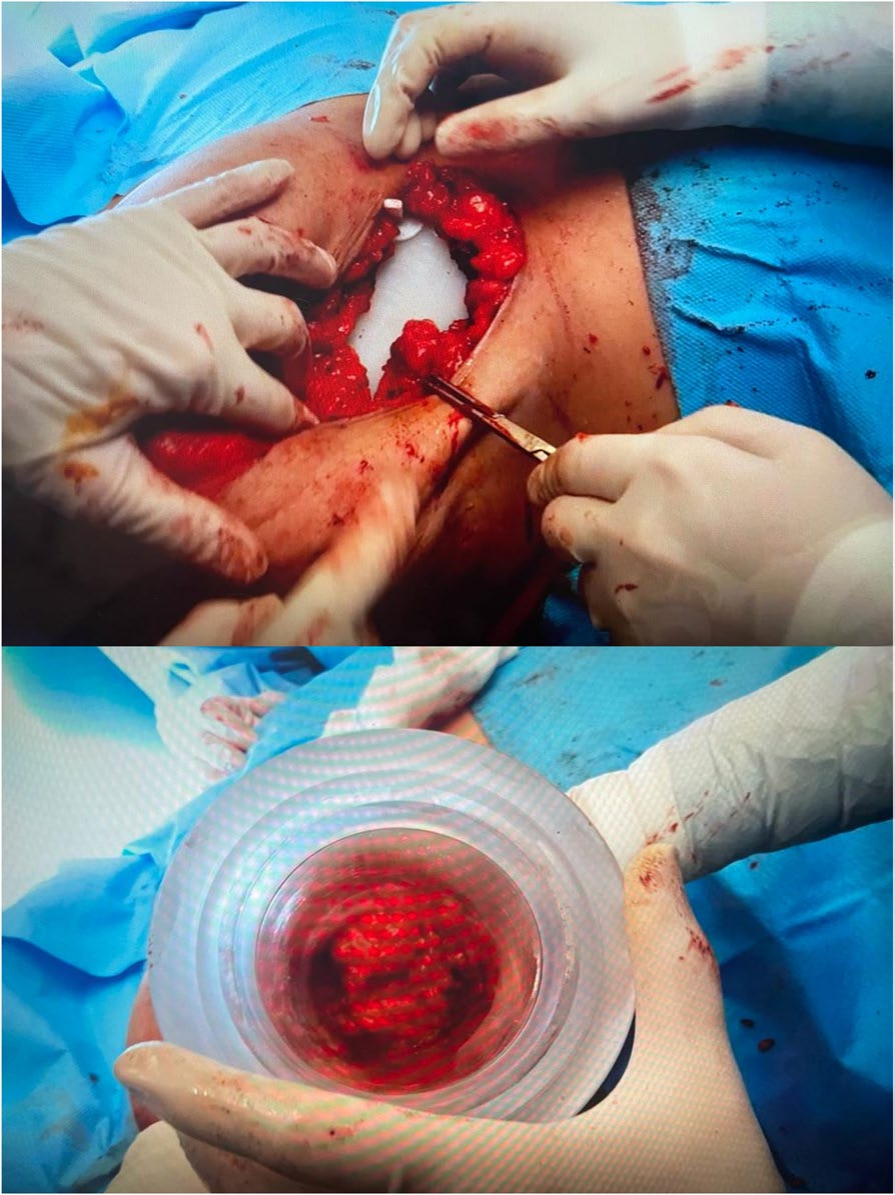
Coverage of marginal tissue by IOERT applicator before radiation

**Fig. 2:**
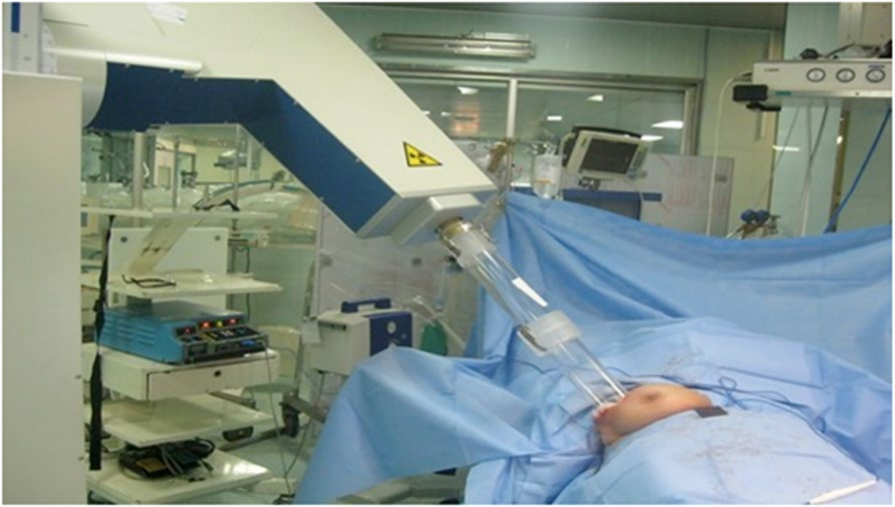
Operation theatre and intraoperative radiation therapy

The LIAC Sordina mobile linear accelerator was used for intraoperative electron beam radiation therapy (IOERT) at Khatam-al-Anbya Hospital, and WBR was performed at Rasoul Akram Hospital. The clinical target volume was determined by the tumor size and site. A dose of 21 Gy and 12 Gy was prescribed to achieve a 95% isodose curve for radical and boosted radiations, respectively. For the preparation of IOERT, the remaining breast tissue was disconnected from the pectoralis major fascia, (Fig. 1) and a lead shield was used to protect the chest wall from irradiation. Also, the breast tissue was detached from the underlying skin to spare the skin and reduce the possibility of skin necrosis. (Fig. 2).

The commencement of radiotherapy for the WBR group was dependent on chemotherapy requirements. For those who received chemotherapy, their WBR sessions began 4–5 months after surgery, and for those who did not receive chemotherapy, their WBR sessions started 3 weeks after surgery. Patients in this group received a fractionated dose of 50 in 32 sessions of WBR.

The intraoperative radiotherapy group was divided into two subgroups (boosted IOERT and radical IOERT). Those in the radical IOERT group (20 patients) received 21 Gy radiotherapy in a surgical setting and those in boosted IOERT (38 patients) received intraoperative boosted electron beam radiotherapy (12 Gy) and a routine fractionated dose of 50 in 25 sessions of WBR. In the boosted IOERT group, WBR sessions were started 4–5 months or 3 weeks after surgery based on the completion of chemotherapy.

### Outcome

In pursuit of a reliable and comprehensive cosmetic scoring system with regard to the geographic features of our study (anatomy and size of the breasts are more varied in Asian women), we designed an objective grading system by combining and modifying other qualitative and objective scoring systems (e.g. Harvard Scale of cosmetic outcome [16], Breast Retraction Assessment (BRA) [17]) for the cosmetic outcomes. Evaluated factors were symmetry in size and shape, discoloration at the surgical site, prominence of scar, deformity, and nipple-areola complex (NAC) position. These characteristics were scored on a three-point scale. With the sum of all factors determining the final cosmetic score. The higher the final score, the better the cosmetic outcome. (Table 1).

**Table 1:** Scoring criteria for the grading system

Cosmetic scoring criteria	Scores		
	0	1	2
**symmetry in size and shape**	An obvious difference in size or shape in comparison to the untreated breast	Slight difference in size or shape on palpation in comparison to the untreated breast	Minimal or no difference in size and shape on palpation in comparison to the untreated breast
**discoloration at the surgical site**	Obvious discoloration of the treated breast	Mild color changes of the treated breast compared to the opposite breast	Color changes are minimal or none
**prominence of scar**	Severe scarring and thickening of the breast skin	Moderate scar tissue causing mild changes in the shape of the breast	Mild thickening or scar tissue without any specific changes in the appearance
**deformity**	Obvious deformation of the breast contour, severe retraction or fibrosis, severe telangiectasia	Mild deformity, localized telangiectasia	Similar appearance without noticeable deformity
**Nipple-areolar complex (NAC) position**	Obvious NAC displacement (≥10 mm)	Mild asymmetry in areola or nipple shape, size or color, Mild NAC displacement (< 10 mm)	NAC is symmetrical

**Fig. 3:**
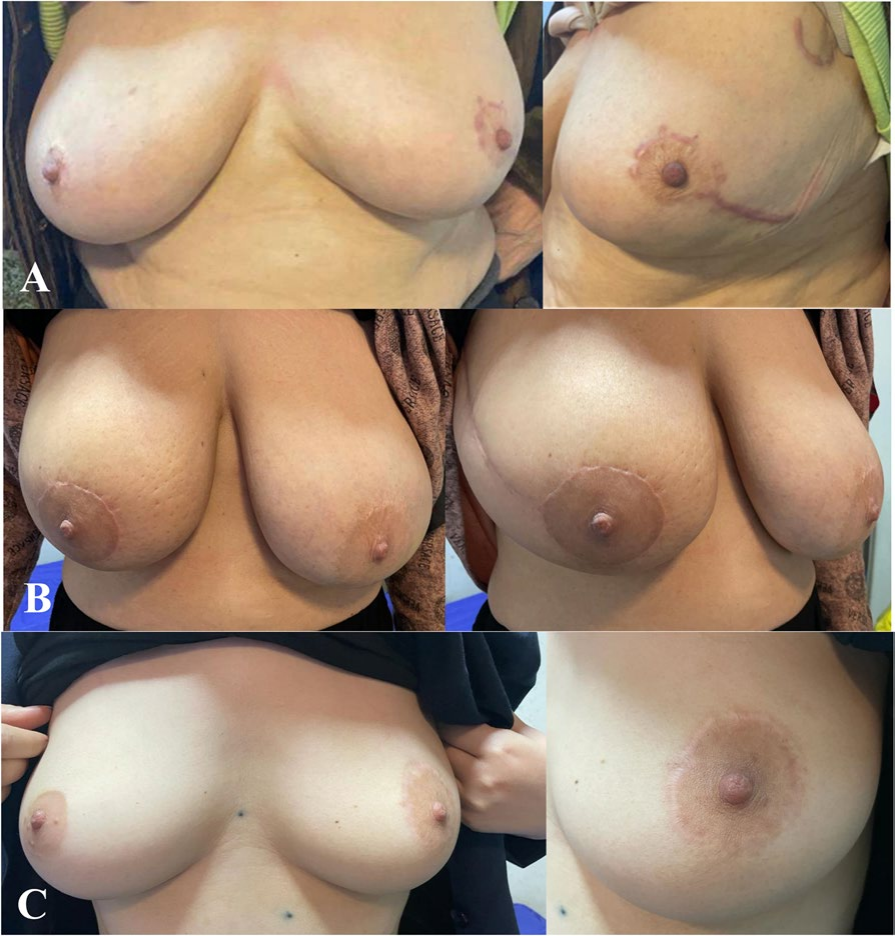
photographs of patients in each group and their cosmetic score; **A** WBR group, cosmetic score: 7/10. **B** Radical IOERT group, cosmetic score: 8/10. **C** boosted IOERT group, cosmetic score 8/10.

A breast surgeon, blinded to the intervention and the patient’s identity, performed a physical examination and documented the cosmetic score. Examples of a patient’s cosmetic outcome and scores are demonstrated in Fig. 3.

Secondary outcomes were the presence of fat necrosis and fibrosis, and postoperative chronic pain. The presence of fat fibrosis and necrosis was evaluated using mammographic findings [18]. The presence of chronic pain was scored using a visual analog scale. Scores of 0 and 1 were defined as negative chronic pain, while 2–10 were labeled as positive chronic pain.

For those who received radical IOERT, outcomes were assessed 6 months after surgery (6 months after IOERT). The boosted IOERT group and the WBR group were each evaluated 6 months after the end of the external beam radiotherapy course (6 months after WBR).

### Statistical analysis

Data analysis was performed using the Statistical Package for Social Sciences 26 (SPSS Inc., Chicago, Illinois, USA). Quantitative variables are expressed as mean ± standard deviation, and for qualitative variables, frequency (%) is used. The chi-squared tests were used to analyze breast involvement, chemotherapy, and endocrine therapy in each group. The pathology in each group was analyzed using the fissure-exact test. For quantitative variables, the Pearson correlation test and the Spearman test were used. Additionally, the Mann–Whitney U test and T-test were used as appropriate. A *p*-value of less than 0.05 was considered statistically significant. Multivariable logistic regressions were conducted by taking all significant covariates in the univariable analysis at a significance level of 25% [19].

## Results

### Patients’ characteristics

From two referral centers, 116 female patients with breast cancer from 2013 to 2018 were enrolled in two groups of 58, determined by the intervention they received. Patients’ ages ranged from 30 to 79 (IOERT patients were 45) and tumor diameters ranged between 0.4 cm and 5 cm. Patients’ characteristics in each intervention group are reported in Table 2.

**Table 2:** Tumor and patients’ characteristics in IOERT and WBR groups

Variables		Total; *N* = 116	Group		***P***-value
			IOERT; *n* = 58	WBR; *n* = 58	
**Age**; mean ± SD		49.24 ± 10.12	51.46 ± 10.46	47.01 ± 9.12	0.170^a^
**Tumor size**; median (IQR)		2.50 (1.50)	2.15 (1.50)	2.50 (1.50)	0.098^d^
**Involved Breast**; n (%)	Right	67 (57.8)	35 (60.3)	32 (55.2)	0.573^b^
	Left	49 (42.2)	23 (39.7)	26 (44.8)	
**Pathology**; n (%)	IDC + DCIS	24 (20.7)	6 (10.3)	18 (31.0)	0.008^c^
	ILC	10 (8.6)	9 (15.5)	1 (1.7)	
	IDC	63 (54.3)	34 (58.6)	29 (50.0)	
	DCIS	11 (9.5)	4 (6.9)	7 (12.1)	
	IDC + ILC	5 (4.3)	3 (5.2)	2 (3.4)	
	IMC	3 (2.6)	2 (3.4)	1 (1.7)	
**Chemotherapy**; n (%)	Yes	45 (38.8)	20 (34.5)	25 (43.1)	0.341^b^
	No	71 (61.2)	38 (65.5)	33 (56.9)	
**Endocrine therapy**; n (%)	Yes	71 (61.2)	38 (65.5)	33 (56.9)	0.446^b^
	No	45 (38.8)	20 (34.5)	25 (43.1)	

*IQR* interquartile range, *DCIS* Ductal Carcinoma in Situ, *IDC* Invasive Ductal Carcinoma, *ILC* Invasive Lobular Carcinoma, *IMC* Invasive medullary Carcinoma, *IOERT* Intraoperative electron Radiation Therapy, *WBR* Whole breast radiotherapy, *SD* Standard deviation

^a^Independent sample t-test

^b^Chi-square test

^c^Fisher’s exact test

^d^Mann Whitney U test

Among 116 patients participating in the study, the median tumor size was 2.50 (1.50). 67 (57.8%) patients had right breast involvement, 49 (42.2%) had left breast involvement, 45 (38.8%) received chemotherapy, and 71 (61.2%) received endocrine therapy. The tumor pathology of the majority of our patients was IDC, with 63 (54.3%), and IDC + DCIS was the next common pathology (20.7%). Post-operative cosmetic outcome scores showed no significant difference between the two groups, and both groups achieved a median of 9 (*P*-Value: 0.199). (Table 3).

**Table 3:** Outcome scores in IOERT and WBR groups

Variables		Total; *N* = 116	Group		*P*-value
			IOERT	WBR	
Cosmetic outcome score; median [Q1 – Q3]		9 [9–10]	9 [9–10]	9 [8–10]	0.199^*^
Post-operative chronic pain	Yes	34 (25.9)	19 (32.8)	15 (25.9)	0.415^†^
	No	82 (70.7)	39 (67.2)	43 (74.1)	
Fat necrosis and fibrosis	Yes	34 (29.3)	25 (43.1)	9 (15.5)	0.001^†^
	No	82 (70.7)	33 (56.9)	49 (84.5)	

*IOERT* Intraoperative Electron Radiation Therapy, *WBR* Whole breast radiotherapy

* Mann-Whitney U test

^†^ Chi-square test

The cosmetic outcome scores had no significant association with breast involvement (*P* = 0.648), chemotherapy (*P* = 0.601), endocrine therapy (*P* = 0.601), method of radiation (*P* = 0.532), pathology (*P* = 0.632), age (*P* = 0.602), and tumor size (*P* = 0.070).

Regarding postoperative chronic pain, no significant difference was reported between the two groups. Based on univariate analysis, post-operative chronic pain had a significant association with tumor size (*P* = 0.022), but no significant association with breast involvement, pathology, chemotherapy, endocrine therapy, method of radiation, and age (*P* = 0.165, 0.179, 0.734, 0.734, 0.415, 0.648, respectively).

Fat necrosis and fibrosis had a significant association with chemotherapy, endocrine therapy, and method of radiation (*P* = 0.044, 0.044, 0.001, respectively), but no significant association with breast involvement, pathology, age, and tumor size (*P* = 0.329, 0.141, 0.981, 0.477, respectively). Fat necrosis and fibrosis had significantly higher rates in the IOERT group in comparison to the WBR group, which was statistically significant as well (P. Value: 0.001), suggesting a higher probability of fat necrosis and fibrosis following IOERT.

Significant covariates in the univariate model were included in the multivariable linear regression. Lower postoperative pain was correlated with smaller tumor size (*P* = 0.013; OR = − 0.341). A higher chance of fat necrosis and fibrosis was correlated with IOERT (*P* = 0.001, OR = 1.601) and chemotherapy (*P* = 0.017, OR = 1.092).

For further investigation, we divided the IOERT group into two subgroups based on whether they received radical IOERT or boosted IOERT plus WBR. 38 (65.5%) of our patients received boosted IOERT plus WBR.

Among the three groups (WBR, boosted IOERT plus WBR, and radical IOERT), there was no significant difference observed regarding cosmetic scores (*P* = 0.315) or post-operative chronic pain (*P* = 0.460), while a significant difference was recorded in fat necrosis and fibrosis (*P* < 0.001). The majority (21/34 or 61.8%) of this complication was observed in the boosted IOERT group.

In a bicategorical evaluation between Radical 21Gy and boosted 12Gy IOERT plus WBR, fat necrosis and fibrosis were significantly higher in the boosted IOERT group (55.3% vs 20.0%; *P* = 0.010). To develop this point further, we recorded that among those who had fat necrosis and fibrosis in the IOERT groups, 84% (21/25) of patients were in boosted IOERT group. However, no significant difference between the WBR and radical IOERT was recorded (*P* value = 0.730).

**Fig. 4:**
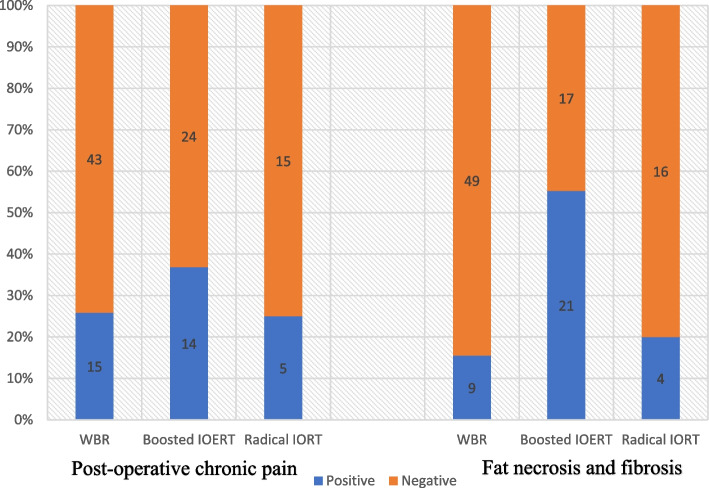
comparison of the post-operative chronic pain and fat necrosis/fibrosis among whole breast radiotherapy (WBR), boosted IOERT, and radical IOERT.

Figure 4 demonstrates the rate of these complications in each group. It is also worth mentioning that there was no significant difference between the WBR and radical IOERT regarding cosmetic scores and post-operative chronic pain. (*P* values of 0.235 and 1.000).

Fat necrosis and fibrosis resulting in wound dehiscence and chronic fistula requiring surgical debridement were observed in two IOERT patients (one in the boosted IOERT subgroup and one in the IOERT subgroup).

## Discussion

Intraoperative radiotherapy was first used in 1998, and since then, many randomized clinical trials have been testing aspects of its effectiveness in terms of treatment. This method, offered as an adjuvant treatment, has been used on over 20,000 women over the years.

TARGIT-A trial demonstrated that the rate of local recurrence of cancer after IORT is not different from external beam radiation therapy, therefore, TARGIT-IORT during lumpectomy is non-inferior to WBR for local recurrence [4, 20]. In a more recent meta-analysis, there were concerns regarding the high local recurrence risk associated with IORT, however, compared to WBR, overall survival, recurrence-free survival, distant metastasis-free survival, and cancer-specific survival was not significantly different. Therefore a proper patient selection strategy should always be applied to identify suitable candidates with a lower risk of local recurrence for IORT [21].

Another aspect of breast cancer treatment is the patient’s experience, quality of life, and minimizing post-therapy complications. The current study aims to compare two radiotherapy methods (IOERT and WBR) in terms of cosmesis and subsequent complications.

Intraoperative radiotherapy has been associated with acceptable results in terms of cosmetic outcomes. In this regard, Kraus et al. evaluated cosmetic outcomes following IORT using the Lent-Soma scale and reported 90% patient satisfaction [22]. In another study conducted by Mi et al. single-dose IORT (20Gy) was used for 77 patients with early-stage breast cancer. Using the Harvard scale, the authors scored 95.9% of patients as excellent or good [23]. Our results in the IOERT group demonstrated a mean score of 9 out of 10, which can be considered “excellent” on the Harvard scale. These findings are in consistent with the mentioned studies.

While WBR has been utilized as a routine method of radiotherapy for years, its adequacy in terms of aesthetic outcome in comparison to more advanced methods has been a controversial subject among researchers [24].

Whelan et al. conducted a study comparing WBR (42.5 Gy in 16 fractions once per day over 21 days, or 50 Gy in 25 fractions once per day over 35 days) with APBI (38.5 Gy in ten fractions delivered twice per day over 5–8 days) in 2135 women and reported similar nurse assessed cosmetic scores at baseline, however, adverse cosmesis (fair or poor) was higher in APBI group at 3, 5 and 7 years. In this study, 36% and 19% of patients were scored fair or poor after 7 years in ABPI and WBR groups, respectively. Although, the authors mentioned that these relatively high rates of adverse cosmesis, might be related to the number of doses administered in the ABPI group [25].

In the Budapest randomized trial by Polgár et al., a ten-year follow-up of 58 women who received either WBR (42–50 Gy/2 Gy per day over 5 weeks) or multi-catheter brachytherapy as ABPI (36.4 Gy/7F), was reported. In this study, the cosmetic score of 81% of patients in the ABPI group was excellent-good while this rate for the conventional WBR group was only 63% (*p*-value < 0.01) [26].

The same favorable cosmetic results were reported in a subset of patients in TARGIT-trial (using the Cosmesis Harris Scale and scored by patients, doctors, nurses, and BCCT.core software) at year 5. In this study, Excellent-good cosmetic outcomes were significant in IORT compared to whole breast external beam radiotherapy, however, across all other time points, results were almost identical [27].

In the long-term (5 year) results of the randomized phase III APBI-IMRT-Florence trial, patients in the APBI group received a dose of 30 Gy in 5 non-consecutive once-daily fraction and those in WBR were administered 50 Gy in 25 fractions followed by a boost dose on the surgical bed (10 Gy in 5 fractions). In contrast to Whelan et al., adverse cosmesis was experienced more in the WBR group in the Florence trial [28]. Although the author of this article mentioned that the method of WBR used might be outdated and the more modern methods (hypofractionated WBR without boost) may result in better cosmetic outcomes.

We used a dose of 50 in 32 sessions in WBR, a single-dose of 21 Gy in the IOERT group, and in boosted IOERT group, we used boosted electron beam radiotherapy (12 Gy) and a dose of 50 Gy in 25 sessions of WBR. Our data showed a median of 9 points in physician-assessed cosmesis for both groups. Our results were mostly in consistent with the study by Whelan et al. and when compared to Budapest and TARGIT trial, patients in our WBR group achieved higher cosmetic results.

When comparing boosted IOERT with the other groups, we did not record a significant difference in terms of cosmesis. This is consistent with the results of the Florence trial and another study by Lemanski et al., which reported mostly good to excellent outcomes in the boosted IORT group. Intensity, technique, and timing between boosted IOERT and the commencement of WBR, reportedly may affect the cosmetic outcome, therefore, further investigation on these factors might be beneficial to improve this method [25, 26].

Precision breast IORT (PB-IORT) developed by Meneveau et al. in 2021 is a more novel form of IORT which uses a multi-catheter brachytherapy balloon to deliver high-dose-rate (HDR) brachytherapy. In this method, an intraoperative CT scan is used to confirm the position of the catheter and adjust the treatment plan. In this form of IORT, 12.5 Gy in a single fraction is delivered to the target volume (1 cm depth) which is significantly higher than the 5–7 Gy used in the conventional breast IORT (the form of IORT used in TARGIT-trial). The cosmetic outcome evaluated using the Harvard scale in this study, demonstrated a 95% “excellent” or “good” score at 12 months post-treatment. Furthermore, only a few patients experienced differences in size, shape, or color between the treated and untreated breasts. Although this study did not focus on comparing all forms of IORT with PB-IORT, it was reported that this method offers better cosmesis compared to conventional breast IORT [29].

Regarding adverse events after therapy, we evaluated the rate of chronic pain, which is an important matter affecting the quality of life. Some studies considered a direct or inverse relationship between this and IOERT [30, 31]. However, no statistically significant relationship between chronic pain and IOERT was observed in our study.

We observed an incident rate of 43.1% of fat necrosis and fibrosis in the IOERT groups and 15.5% in the WBR group. This finding is in agreement with other studies [32–34]. In a meta-analysis by Wang et al., the risk of skin toxicity was reported significantly lower in the IORT group, however, fat toxicity incident was 3.106 times higher compared to the WBR group [21]. In the report of the late side-effects and cosmetic outcomes of the phase III GEC-ESTRO trial, The risk of developing symptomatic fat necrosis in 5 years did not differ between APBI and WBR groups. However, the risk of developing grade 2 and 3 late subcutaneous tissue side-effects (based on the Radiation Therapy Oncology Group/European Organisation for Research and Treatment of Cancer Late Radiation Morbidity Scoring Schema) at 5 years was 9·7% (95% CI 7·1–12·3) and 12·0% (9·4–14·7%) for WBR and APBI, respectively.

In our study, the rate of fat necrosis and fibrosis was more significant in the boosted IOERT subgroup (55.3%). Among those who had fat necrosis and fibrosis in the IOERT groups, 84% of patients were in boosted IOERT group which received 12Gy IOERT and 25 seasons of WBR. However, in this regard, no significant difference between the WBR and radical IOERT was recorded. In this regard, Lemanski et al. reported a 14% incidence of subcutaneous fibrosis in the surgical area [35] and in another study, grade III fibrosis was observed in two out of twenty-four women observed [36]. We believe that the high rate of fat necrosis and fibrosis in the setting of boosted IOERT in our study is mainly because these patients received radiotherapy twice (boosted IOERT and WBR). Based on these findings, further studies on comparing boosted IOERT to other forms of IOERT with groups matched in their baseline data and a focus on fat necrosis and fibrosis are recommended.

One of the strengths of our study is that it was performed in Asia, where the anatomy and size of the breasts are more varied in women [23]. The limitations of our work can be described as limited data, a short follow-up period, using a new scoring system which, although adapted from the Harvard scale, had not been validated and it can be stated that there might be confounding factors that may change the result of our work to some extent. Therefore, additional studies with a larger sample size are required to validate the findings of this study.

## Conclusion

The IOERT in breast cancer treatment is a modern method, which is still considered an experimental treatment option. In our study, this method was associated with similar cosmetic outcomes and no significant difference in complications compared to the traditional WBR.

## Data Availability

The datasets used and/or analyzed during the current study are available from the corresponding author on reasonable request and with permission of the Research Ethics Committee of the School of Medicine-Tehran University of Medical Sciences.

## Abbreviations

BCT: Breast-conserving therapy
WBR: Whole breast radiotherapy
APBI: Accelerated partial breast irradiation
IOERT: Intraoperative electron radiation therapy
AJCC: American Joint Committee on Cancer
NAC: Nipple-areola complex
IDC: Invasive Ductal Carcinoma
ILC: Invasive Lobular Carcinoma
LCIS: Lobular carcinoma in situ
DCIS: Ductal Carcinoma in Situ
ER: Estrogen receptor
IMC: Invasive medullary Carcinoma
SD: Standard deviation
TARGIT: Targeted intraoperative radiotherapy
